# Challenges of single-stage pancreatoduodenectomy: how to address pancreatogastrostomies with robotic-assisted surgery

**DOI:** 10.1007/s00464-021-08925-w

**Published:** 2021-12-09

**Authors:** Lea Timmermann, Karl Herbert Hillebrandt, Matthäus Felsenstein, Moritz Schmelzle, Johann Pratschke, Thomas Malinka

**Affiliations:** 1 Department of Surgery, Charité-Universitätsmedizin Berlin, Corporate Member of Freie Universität Berlin, Humboldt-Universität zu Berlin, and Berlin Institute of Health, Humboldt-Universität zu Berlin, Berlin, Germany; 2 Berlin Institute of Health, Berlin, Germany; 3 Department of Surgery, Charité Campus Mitte and Charité Campus Virchow Klinikum, Charité Universitätsmedizin Berlin, Campus Virchow-Klinikum, Augustenburger Platz 1, 13353 Berlin, Germany

**Keywords:** Pancreatogastrostomy, Pancreato-enteric anastomosis, Pancreatoduodenectomy, Robotic-assisted

## Abstract

**Introduction:**

Establishing a sufficient pancreatico-enteric anastomosis remains one of the most important challenges in open single stage pancreatoduodenectomy as they are associated with persisting morbidity and mortality. Applicability on a robotic-assisted approach, however, even increases the requirements. With this analysis we introduce a dorsal-incision-only invagination type pancreatogastrostomy (dioPG) to the field of robotic assistance having been previously proven feasible in the field of open pancreatoduodenectomy and compare initial results to the open approach by means of morbidity and mortality.

**Methods:**

An overall of 142 consecutive patients undergoing reconstruction via the novel dioPG, 38 of them in a robotic-assisted and 104 in an open approach, was identified and further reviewed for perioperative parameters, complications and mortality.

**Results:**

We observed a comparable R0-resection rate (*p* = 0.448), overall complication rate (*p* = 0.52) and 30-day mortality (*p* = 0.71) in both groups. Rates of common complications, such as postoperative pancreatic fistula (*p* = 0.332), postoperative pancreatic hemorrhage (*p* = 0.242), insufficiency of pancreatogastrostomy (*p* = 0.103), insufficiency of hepaticojejunostomy (*p* = 0.445) and the re-operation rate (*p* = 0.103) were comparable. The procedure time for the open approach was significantly shorter compared to the robotic-assisted approach (*p* = 0.024).

**Discussion:**

The provided anastomosis appeared applicable to a robotic-assisted setting resulting in comparable complication and mortality rates when compared to an open approach. Nevertheless, also in the field of robotic assistance establishing a predictable pancreatico-enteric anastomosis remains the most challenging aspect of modern single-stage pancreatoduodenectomy and requires expertise and experience.

Reconstruction techniques following pancreatoduodenectomy (PD) are almost as old as the resection itself. In 1946, Whipple was first to perform an open single stage PD (OPD) and established restoration via pancreaticojejunostomy (PJ) [1]. Today, in the field of OPD, two main types of pancreatico-enteric anastomoses are applied. On the one hand, PJ is widely performed as a two-layered suture consisting of a duct-to-mucosa and a parenchyma-to-wall anastomosis, referred to also as the Blumgart or modified Blumgart-anastomosis [2]. On the other hand, there is pancreatogastrostomy, which Waugh and Clagett initially performed in 1944 [3]. It is commonly performed as an invagination type rather than a duct-to-mucosa anastomosis. Over the past decades, generations of pancreatic surgeons have made several modifications and advocated slight advantages of either PJ or PG [4, 5]. Comprehensive analysis, however, did not find significant differences [6, 7]. The establishment of a pancreatico-enteric anastomosis is still associated with the most common threats of OPD. Postoperative pancreatic fistula (POPF), postoperative pancreatic hemorrhage (PPH), or intraabdominal abscesses may result in severe morbidity and, consecutively, persistent perioperative mortality.

However, the holy grail of predictable restoration of the pancreatic remnant following OPD has not been found yet. Several risk factors for the appearance of POPF have been proposed, commonly including soft tissue texture and a narrow-calibered pancreatic duct [8]. Over the past years, minimally invasive procedures became popular in the field of pancreatic surgery. As limitations of laparoscopic techniques for such complex procedures are eminent, they remain an appropriate approach mainly for distal pancreatectomies. On the other hand, a robotic-assisted approach allows for up to seven degrees of freedom, a three-dimensional view [9] and reduces tremor transmission [10]. Therefore, it seems suitable for resection and complex reconstructions following pancreatoduodenectomy (RPD).

Nevertheless, the transferability of established techniques for pancreatico-enteric anastomoses to a robotic-assisted approach still lacks evidence. The learning curve for robotic-assisted pancreatic surgery is significantly shorter than for laparoscopy [11, 12]. Several studies confirmed its safety compared to open procedures in terms of common complications [13–15]. We recently established a new dorsal-incision-only invagination type pancreatogastrostomy (dioPG) following OPD, which we now implemented for robotic-assisted procedures. This study presents our first experiences with the dioPG following RPD and compares the results to our first consecutive patients undergoing dioPG following OPD for outcome parameters such as perioperative mortality and complication rates.

## Methods

### Data collection and exclusion criteria

We conducted a prospective single-centre observational study at our tertiary referral centre for pancreatic surgery to analyze perioperative outcome parameters of robotic-assisted pancreatic surgery using the da Vinci Xi surgical system (Intuitive, Sunnyvale, CA, USA). Data of all consecutive patients who underwent PD with restoration of the pancreatic remnant via dioPG between October 2018 and December 2020 were collected within the CARE-Study (surgical assistance by robotic support; originally Chirurgische Assistenz durch Robotereinsatz, ethical approval code E/A4/084/17; (DRKS00017229)). Data of all consecutive patients, who underwent PD with restoration of the pancreatic remnant via dioPG between October 2018 and December 2020 in an open approach, were retrospectively analyzed and compared to those having undergone a robotic approach. Patients undergoing other resections, including distal and total pancreatectomies, or reconstruction via PJ or classic pancreatogastrostomy requiring a ventral gastrotomy (vgPG) were excluded from further analysis. Eleven patients undergoing RPD were excluded because reconstruction was carried out via the retrieval incision in a hybrid procedure during the program’s implementation phase. We included an overall of 142 cases—104 cases with OPD and 38 cases with RPD. We included the following data: age, sex, preoperative ASA-score, preoperative BMI, R0-resection state, operation time, overall complications, Clavien/Dindo classification, POPF, PPH, DGE, PG-insufficiency, BDA-insufficiency, surgical site infections (SSI), reoperation rate, intervention rate, in-hospital stay, 30-day mortality and 90-day readmission rate. POPF, PPH and DGE were defined and classified after the International Study Group of Pancreatic Surgery (ISGPS) classifications [16–18].

### Preoperative assessment and preparation

The preoperative assessment followed a standardized schedule, including a physical examination, laboratory testing and anesthesiological evaluation. Either computed tomography with contrast agents or magnetic resonance imaging indicated resectability and confirmed given indication. In cases of underlying malignancy, an interdisciplinary tumour board evaluated each case after completed staging, including, e.g. endosonography and chest imaging.

### Surgical approach

**Fig. 1:**
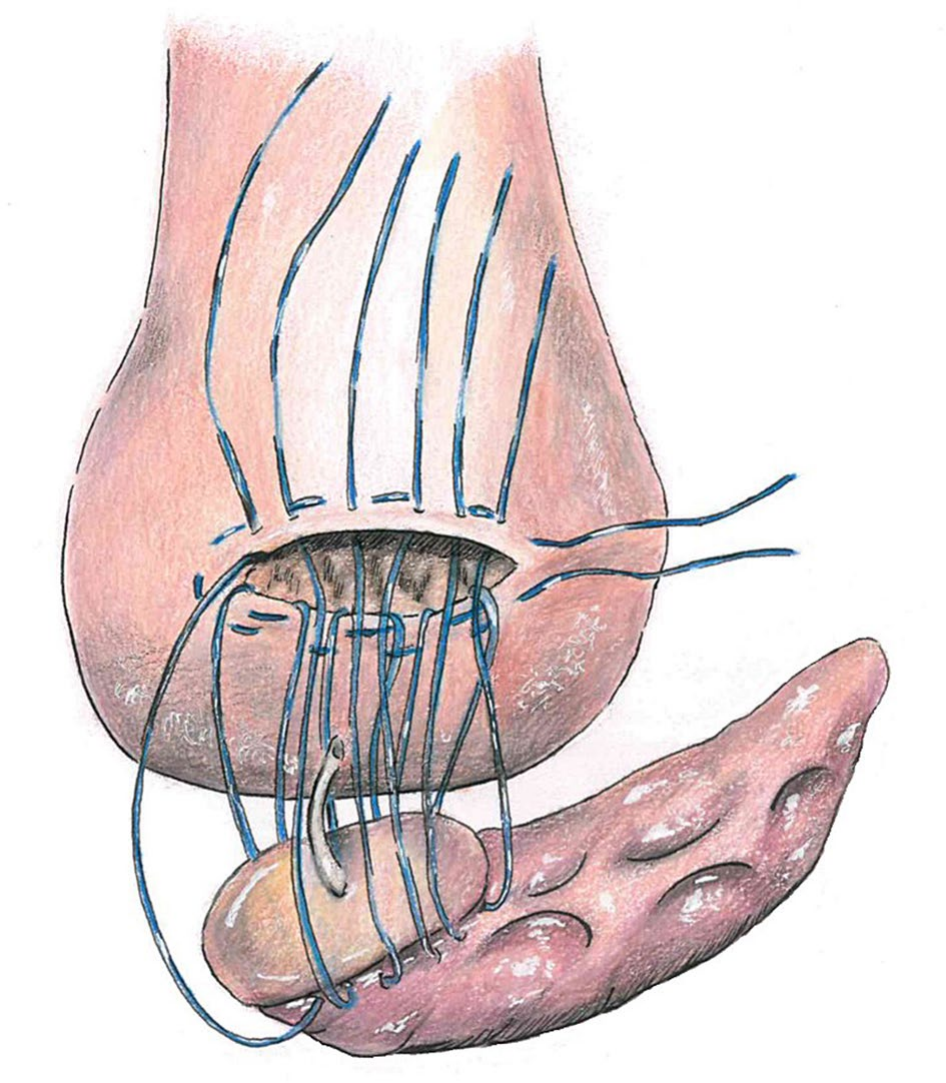
dioPG in progress. Figure shows the anastomosis in progress with the untied purse-string and mattress sutures

We used the da Vinci Xi surgical system for all RPDs. Electrocautery was deployed for dissection of the pancreas in both approaches. Our modified dioPG was performed as follows: after an oblique incision to the posterior gastric wall, a purse-string suture (Prolene® 4/0) was placed around the cut surface without instantly tying. Afterwards, three to four mattress sutures (double armed PDS® 4/0, MH1 needle) were placed through either side of the cut surface of the posterior gastric wall and the pancreatic remnant in between. Tying these mattress sutures leads to luxation of the stump through the posterior gastric wall. After tying the mattress sutures, the purse-string suture is then tied in the final step. In the case of a robotic-assisted approach, the retrieval incision served for haptic reevaluation of the implemented anastomosis. Figure 1 shows the anastomosis in progress with the untied purse-string and mattress sutures.

### Postoperative course

Following an intensive care unit stay of at least one day post-surgery, patients passed to our surgical ward. The standardized course included a daily examination, laboratory testing and measuring of drainage lipase levels. If laboratory testing ruled out POPF, drainages were commonly removed on day three after surgery. An X-ray swallow study examined PG insufficiency and signs for gastric emptying disorder on day five after surgery. After removing the nasogastric tube, oral food intake was permitted.

### Statistics

Data were processed using SPSS version 25.0 (IBM, Armonk, NY, USA). Two-tailed Pearson’s chi-square test and Fisher’s exact test were performed on categorical and ordinal scaled data, and student’s t-test was performed on interval scaled data. Significance tests were two-sided, and *p* < 0.05 was considered to be statistically significant.

## Results

### Patients’ characteristics

We included an overall of 142 patients for further analysis. Thirty-eight underwent reconstruction via dioPG with a robotic-assisted approach and 104 in an open approach from October 2018 to December 2020. The patient’s baseline characteristics were comparable in both groups apart from age. Patients in the RPG group were significantly younger (61.5 years) than patients in the OPG group (68.1 years; *p* = 0.002). Patients in the RPG group had significantly fewer malignant diseases (*p* = 0.002) compared to the OPG group. PDAC was more often an indication for surgery in the OPG group, whereas extrahepatic cholangiocellular carcinoma was more often an indication for surgery in the RPG group. In cases of underlying malignancy, T-stage was slightly higher in the OPG group, although this finding did not reach statistical significance. The tumor diameter, however, was significantly higher in the OPG group (*p* = 0.0002).

Table 1 indicates patients characteristics.

**Table 1:** Patients’ characteristics

Characteristics *N* (%)	All patients (*N* = 142)	RPG (*N* = 38)	OPG (*N* = 104)	*p* value
Sex *N* (%)				0.431
Male	82 (57.7)	21 (55.3)	61 (58.7)	
Female	60 (42.3)	17 (44.7)	43 (41.3)	
Age (years)				
Mean	66.4	61.5	68.1	**0.002**
Minimum	37	37	38	
Maximum	88	78	88	
ASA score *N* (%)				0.231
1	4 (3)	0 (0)	4 (4)	
2	67 (49.6)	18 (51.4)	49 (49)	
3	63 (46.7)	16 (45.7)	47 (47)	
4	1 (0.7)	1 (2.9)	0 (0)	
BMI *N* (%)				0.931
Mean	25.1	24.9	25.08	
Minimum	17	17	17	
Maximum	42	40	42	
Underlying malignancy *N* (%)	108 (76.1)	22 (57.9)	86 (82.7)	*0.002*
Indication *N* (%)				
PDAC	62 (43.7)	10 (26.3)	52 (50)	
Extrahepatic cholangiocarcinoma	18 (12.7)	6 (15.8)	12 (11.5)	
Papillary carcinoma	13 (9.2)	5 (13.2)	8 (7.7)	
Duodenal carcinoma	3 (2.1)	1 (2.6)	2 (1.9)	
GIST	5 (3.5)	0 (0)	5 (4.8)	
Other (malignant)	7 (4.9)	0 (0)	7 (6.7)	
Pancreatitis	17 (12)	6 (15.8)	11 (10.6)	
IPMN	11 (7.7)	7 (18.4)	4 (3.8)	
Other (benign)	6 (4.2)	3 (7.9)	3 (2.9)	
T-stage *N* (%)				0.096
T1	16 (15.5)	6 (27.3)	10 (12.3)	
T2	48 (46.6)	12 (54.5)	36 (44.4)	
T3	31 (30.1)	4 (18.2)	27 (33.3)	
T4	8 (7.8)	0 (0)	8 (9.9)	
Tumor diameter (mm)				*0.0002*
Mean	28	19	30.5	
Minimum	1	6	1	
Maximum	120	34	120	

Table indicated baseline characteristics of patients receiving either open or robotic-assisted pancreatoduodenectomy

*ASA* American Society of Anesthesiologists, *BMI* Body Mass Index, *PDAC* pancreatic ductal adenocarcinoma, *GIST* gastrointestinal stroma tumor, *IPMN* intraductal papillary mucinous neoplasm

### Perioperative parameters

The histopathological resection state was comparable in both groups (*p* = 0.448), with R0-resection rates of 74.4% in the OPG group and 86.4% in the RPG group. Operation time was significantly shorter in the OPG group with 263.1 min (134–437 min) compared to the RPG group with 286.2 min (210–382 min; *p* = 0.024).

### Complications

Neither the overall complication rates nor the 30-day mortality rate differed significantly in both groups. We observed an overall complication rate of 66.3% in the OPD group and 60.5% in the RPD group (*p* = 0.52). The 30-day mortality rate in the OPD group was 3.8% and in the RPD group 5.3% (*p* = 0.71), whereas the overall mortality (Clavien/ Dindo 5) was 4.8% in the OPD and 7.9% in the RPD group. Three patients in the OPD group died due to cardiac arrest, one due to a severe septic shock following celiac axis occlusion and one due to hypoglycaemia. In the RPD group, one patient died due to an acute respiratory distress syndrome (ARDS), one due to severe bleeding and one due to transfusion-related acute lung insufficiency (TRALI). The rates of common complications such as POPF, PPH, DGE, SSI and insufficiency of the hepaticojejunostomy were comparable in both groups. We observed more POPF grade B in the RPD than in the OPD group (21.1% vs 13.5%), although this finding did not show statistical significance (*p* = 0.103). The re-operation rate in the RPD group also appeared to be higher (18.4%), although not statistically significant (*p* = 0.103), compared with the OPD group (8.7%). Two patients in the OPD group underwent operative revision of the hepaticojejunostomy, one due to fascia dehiscence, one due to an intraabdominal haematoma, one underwent reconstruction of the celiac axis, one underwent completion pancreatectomy, one underwent splenectomy after CPR, and two underwent reoperation due to surgical site infection. Three patients in the RPD group underwent completion pancreatectomy, two underwent operative revision of the implemented PG, one underwent open herniotomy, and one was treated with a vacuum sealing due to SSI. Completion pancreatectomy in all cases was performed due to persisting pancreatitis and sepsis and (partial) insufficiency to the implemented PG. In two cases of the RPD group, pancreatitis appeared to be necrotizing. Operative revision of the implemented PG was performed due to partial PG-insufficiency in the early postoperative phase without pancreatitis.

Perioperative parameters are shown in Table 2.

**Table 2:** Perioperative parameters and complications

Characteristics *N* (%)	All patients (*N* = 142)	RPG (*N* = 38)	OPG (*N* = 104)	*p* value
R0 resection state *N* (%)	80 (76.9)	19 (86.4)	61 (74.4)	0.448
Operation time (min)				
Mean	269.3	286.2	263.1	***0.024***
Minimum	134	210	134	
Maximum	437	382	437	
Overall complications *N* (%)	92 (64.8)	23 (60.5)	69 (66.3)	0.520
Clavien/dindo classification *N* (%)				0.260
0	49 (34.5)	14 (36.8)	35 (33.7)	
1	9 (6.3)	1 (2.6)	8 (7.7)	
2	14 (9.9)	3 (7.9)	11 (10.6)	
3a	36 (25.4)	11 (28.9)	25 (24)	
3b	7 (4.9)	1 (2.6)	6 (5.8)	
4a	17 (12)	3 (7.9)	14 (13.5)	
4b	2 (1.4)	2 (5.3)	0 (0)	
5	8 (5.6)	3 (7.9)	5 (4.8)	
POPF *N* (%)				0.332
Biochemical leak	3 (2.1)	0 (0)	3 (2.9)	
B	22 (15.5)	8 (21.1)	14 (13.5)	
C	0 (0)	0 (0)	0 (0)	
PPH *N* (%)				0.242
A	9 (6.3)	1 (2.6)	8 (7.7)	
B	10 (7)	2 (5.3)	8 (7.7)	
C	5 (3.5)	3 (7.9)	2 (1.9)	
SSI *N* (%)	13 (9.2)	3 (7.9)	10 (9.6)	0.753
DGE *N* (%)	13 (9.2)	4 (10.5)	9 (8.7)	0.732
PG-insufficiency *N* (%)	16 (11.3)	7 (18.4)	9 (8.7)	0.103
Insufficiency hepaticojejunostomy *N* (%)	7 (4.9)	1 (2.6)	6 (5.8)	0.445
Reoperation rate *N* (%)	16 (11.3)	7 (18.4)	9 (8.7)	0.103
Intervention *N* (%)	53 (37.3)	15 (39.4)	38 (36.5)	0.250
30-day mortality *N* (%)	6 (4.2)	2 (5.3)	4 (3.8)	0.710
90-day readmission rate *N* (%)	17 (12)	3 (7.9)	14 (13.5)	0.366
In-hospital stay (days)				
Mean	18.08	18.9	17.76	0.617
Minimum	3	3	3	
Maximum	68	68	59	

Table compares perioperative complications and outcome parameters for patients receiving either open or robotic-assisted pancreatoduodenectomy

*POPF* postoperative pancreatic fistula, *PPH* postoperative pancreatic hemorrhage, *SSI* surgical site infection, *DGE* delayed gastric emptying, *PG* pancreatogastrostomy

## Discussion

The implementation of a reliable pancreatico-enteric anastomosis, regardless of an open or minimally invasive approach, is the holy grail of pancreatic surgery. Life-threatening complications such as POPF, PPH and intraabdominal abscesses may occur and result in severe morbidity and mortality [7]. While the incidence of POPF decreased over the last decades, its related mortality remains at a range of around 1%. However, it may increase up to 40–50% for grade C fistula [7]. Tissue injuries around the cut surface, traumatic needle channels or cutting suture surfaces [19] as well as tension to the established anastomosis and blood circulation [20] may all impair the sufficiency of the applied pancreatico-enteric anastomosis. Technical challenges of robotic-assisted procedures may now add another factor. The transferability of an anastomosis, whose feasibility has already been proven in OPD, to a robotic-assisted approach is an essential point to be considered. We recently published our first experiences with robotic-assisted procedures in the field of pancreatic surgery [21]. An essential point in procedures performed on an organ with such a variable and often soft texture that instantly influences the success rate of implemented anastomoses and consecutively perioperative morbidity and mortality remains haptic feedback. Therefore, in the first RPD cases, reconstruction (hepaticojejunostomy, pancreatogastrostomy and gastroenterostomy) was performed via the retrieval incision. Although those cases were excluded from the present analysis, we consider the retrieval incision inevitable for sufficient haptic feedback. The evidence for pancreatico-enteric anastomoses following RPD is even less than in OPD, as only a few reports can be found [22–24]. In a published multi-centre series, minimally invasive one-row pancreaticojejunostomy appeared inferior to pancreaticojejunostomy in an open approach. The authors consider an association with the learning curve in progress during this analysis [25]. The implemented dioPG seemed to be a feasible technique in RPD setting as it allows a better exposition of the surgical site and an increased range of motion compared with the vgPG and thereby likely may decrease tissue trauma. During our first experiences with open dioPG, we additionally experienced a decrease in procedure time and rate of DGE compared to open vgPG [26]. In this current series, RPD procedures took significantly longer than OPD procedures as they include patients from our initial learning curve for robotic-assited pancreatic surgery in general. We recently showed a significant decrease in procedure time in RPD after the initial cases, that are also included in this study. The tumor diameter and the rate of underlying malignancy was significantly higher in the OPD group. This could be explained with the implementation phase of robotic assistance where tumors with larger diameter rather underwent open resection. Tumor extent, however, did not influence the technical aspects of the implemented anastomosis.

Compared to open dioPG, we saw no significant difference in perioperative complications after robotic-assisted dioPG. The rate of PG-insufficiency was higher in the RPD group, although these findings did not reach statistical significance,. As the OPD was the standard procedure over the last decades, the higher incidence of PG-insufficiency in the RPD group may have occurred due to the learning curve in progress during these first cases. Overall, the rate of PG-insufficiencies appears high in our cohort. This finding, in addition to the learning curve in progress for the RPD group of this study, might appear due to a change of management consisting of early endoscopy revealing also small insufficiencies that might have been overseen without endoscopy.We furthermore observed a slightly higher reoperation rate in the RPD group, including two patients undergoing PG-revision in the early implementation phase. The implemented anastomosis considers that an invagination type PG entirely covers the cut surface, including the proximal amount of tissue and, thereby, reduces trauma inflicted by sutures and needle channels. The implemented dioPG appears to be a technically feasible option for reconstruction following OPD and RPD. It allows an increased range of motion and a better exposition of the surgical site, especially in RPD. As this study includes cases from the initial implementation phase of robotic-assisted pancreatic surgery in our clinic, further studies are mandatory to proof our initial findings. We believe that haptic reevaluation of established anastomoses remains an essential tool and may improve the patient’s safety.

This study is limited by common biases, mainly due to its retrospective character. When comparing the groups, although the RPD group was significantly younger, they were homogeneous by means of preoperative parameters (gender, BMI and ASA score). Future analyses are mandatory to further evaluate the applicability of the presented anastomosis on robotic-assisted procedures.

## Conclusion

In conclusion, the implementation of a reliable pancreatico-enteric anastomosis remains an issue associated with some of the most common threats in PD. The robotic-assisted procedure furthermore increases the demands on an anastomosis to be established. With this study, we provide a technically feasible technique for the restoration of the pancreatic remnant, which is also applicable to a robotic-assisted approach.
